# Evaluating authentication options for mobile health applications in younger and older adults

**DOI:** 10.1371/journal.pone.0189048

**Published:** 2018-01-04

**Authors:** Kelly Grindrod, Hassan Khan, Urs Hengartner, Stephanie Ong, Alexander G. Logan, Daniel Vogel, Robert Gebotys, Jilan Yang

**Affiliations:** 1 School of Pharmacy, University of Waterloo, Ontario, Canada; 2 Cheriton School of Computer Science, University of Waterloo, Ontario, Canada; 3 University Health Network, Toronto, Ontario, Canada; 4 Department of Psychology, Wilfrid Laurier University, Waterloo, Ontario, Canada; King Saud University, SAUDI ARABIA

## Abstract

**Objective:**

Apps promoting patient self-management may improve health outcomes. However, methods to secure stored information on mobile devices may adversely affect usability. We tested the reliability and usability of common user authentication techniques in younger and older adults.

**Methodology:**

Usability testing was conducted in two age groups, 18 to 30 years and 50 years and older. After completing a demographic questionnaire, each participant tested four authentication options in random order: four-digit personal identification number (PIN), graphical password (GRAPHICAL), Android pattern-lock (PATTERN), and a swipe-style Android fingerprint scanner (FINGERPRINT). Participants rated each option using the Systems Usability Scale (SUS).

**Results:**

A total of 59 older and 43 younger participants completed the study. Overall, PATTERN was the fastest option (3.44s), and PIN had the fewest errors per attempt (0.02). Participants were able to login using PIN, PATTERN, and GRAPHICAL at least 98% of the time. FINGERPRINT was the slowest (26.97s), had an average of 1.46 errors per attempt, and had a successful login rate of 85%. Overall, PIN and PATTERN had higher SUS scores than FINGERPRINT and GRAPHICAL. Compared to younger participants, older participants were also less likely to find PATTERN to be tiring, annoying or time consuming and less likely to consider PIN to be time consuming. Younger participants were more likely to rate GRAPHICAL as annoying, time consuming and tiring than older participants.

**Conclusions:**

On mobile devices, PIN and pattern-lock outperformed graphical passwords and swipe-style fingerprints. All participants took longer to authenticate using the swipe-style fingerprint compared to other options. Older participants also took two to three seconds longer to authenticate using the PIN, pattern and graphical passwords though this did not appear to affect perceived usability.

## Introduction

Privacy is one of the biggest factors that bring down consumer ratings of mobile apps. [1] For mobile health (mHealth) apps, the main privacy concerns are around the leak of stigmatizing information such as sensitive medical diagnoses, test results and medication lists [2]. Yet, most mHealth apps used by consumers do not fall under federal or regional health privacy laws, even when the apps are used to manage a chronic illness [3]. The lack of oversight means consumer mHealth apps are often built without basic security measures to protect against privacy and security breaches including authentication (e.g., passwords), encryption, and up-to-date privacy policies [4,5].

The poor attention to privacy and security is holding back the adoption of useful apps in healthcare. In 2013, for example, the National Health Service (NHS) developed the NHS Health Apps Library formulary to encourage clinicians to prescribe high quality apps. However, the website was taken down two years later after researchers raised several privacy concerns, including that only one in four apps had a username-password or PIN feature—only half of which stored the credentials securely [6]. Similar concerns also led to the suspension of the Happtique health app certification program between 2013 to 2016 [7].

The security shortcomings of mHealth apps are further compounded by the fact that up to 60% of smartphone users do not personally secure their phones [8,9]. From a usability perspective, the lack of security features makes sense as users do not want to be slowed down by a password. However, health data is no less private than financial data—and mobile banking apps typically require a username and/or password. Further, financial apps are much more likely to be used by younger users [10]. In contrast, health apps are designed for older people who have complex health needs, and both age and illness may limit their ability to use standard usernames and passwords [11,12]. Therefore, app developers should at least give users options to secure an mHealth app especially in cases where the user does not want to secure the entire phone.

In 2016, Morera *et al*. published several useful security recommendations for mHealth developers. For “high security level” apps including those for monitoring, diagnosis and treatment, recommended strategies included, but were not limited to, using password or biometric authentication, two-step authentication, and terminating the session after 15 minutes [13]. In practice, these recommendations translate into the following options for developers: something-users-know (a 4-digit personal identification number (PIN) or password), something-users-are (biometrics such as fingerprint, iris or facial recognition) or something-users-have (smart watch or a Bluetooth device). Two-step authentication refers to the use of two of these options (e.g., password and smart watch).

In terms of consumer preference for different authentication options, Bhagavatula *et al*. have shown that most individuals perceive a fingerprint as more convenient than a 4-digit PIN, and that a PIN is preferred over facial recognition [14]. While De Luca *et al*. reported similar findings [15], the focus of both studies was younger adults. In contrast, research by Vu *et al*. on passwords for online services found that older adults are more likely to forget text-based passwords and that graphical password mnemonic techniques can be a useful cue [16]. However, similar research has not been done on authentication strategies for mHealth apps used by older adults. Thus, it is important for mHealth developers to understand how older adults experience authentication options rather than solely relying on research with younger populations or online platforms.

In this study, we put together an interdisciplinary team of health and computer science researchers whose aim is to improve the adoption of safe and secure mHealth apps. Our goal was to help developers choose between the different options for securing mHealth apps. We used methods common to security research, which typically focuses on samples of younger users, and expanded the methods to a population (e.g., older adults) that is more representative of the users in healthcare. Thus, the objective of this multidisciplinary study was to test the reliability and usability authentication strategies with older adults who represent the typical healthcare user and compare their test results to those of younger individuals.

## Methods

The Clinical Research Ethics Board at the University of Waterloo, Waterloo, Ontario, Canada approved the study protocol and all participants gave informed consent. In this study, we tested four authentication interfaces: four-digit PIN, graphical password, pattern-lock, and fingerprint on an Android-based mobile application. We did not include complex text passwords (e.g., eight characters including a number, letter and/or symbol), as they are difficult to enter on touch keyboards. We also did not include facial recognition as it requires good lighting conditions, the ability to align the device camera with the face and it has not been fully proven for smartphone use. Participants who had previous experience with knowledge-based authentication measures were encouraged to create sequences that they had not used previously.

### Setting and participants

We tested the authentication techniques in two age groups, 18 to 30 years and 50 years and older. We hypothesized that older adults would have greater age- and disease-related variability and we recruited more older adults to account for this. To be eligible for the study, participants had to be able to speak and read English and to have prior experience using a smartphone or tablet. All participants completed the Health Literacy Assessment (HLA) [17] to assess health literacy and the Montreal Cognitive Assessment (MoCA) [18] to assess cognitive function.

Older adults were recruited through public libraries, senior education sessions, senior computer clubs and community centers. Younger adults were recruited through university undergraduate programs and online through the buy-and-sell website Kijiji.ca. Research interviews were conducted at the university, in participant homes and in coffee shops according to participant preference, and participants were given a $10 honorarium.

### Intervention

**Fig 1:**
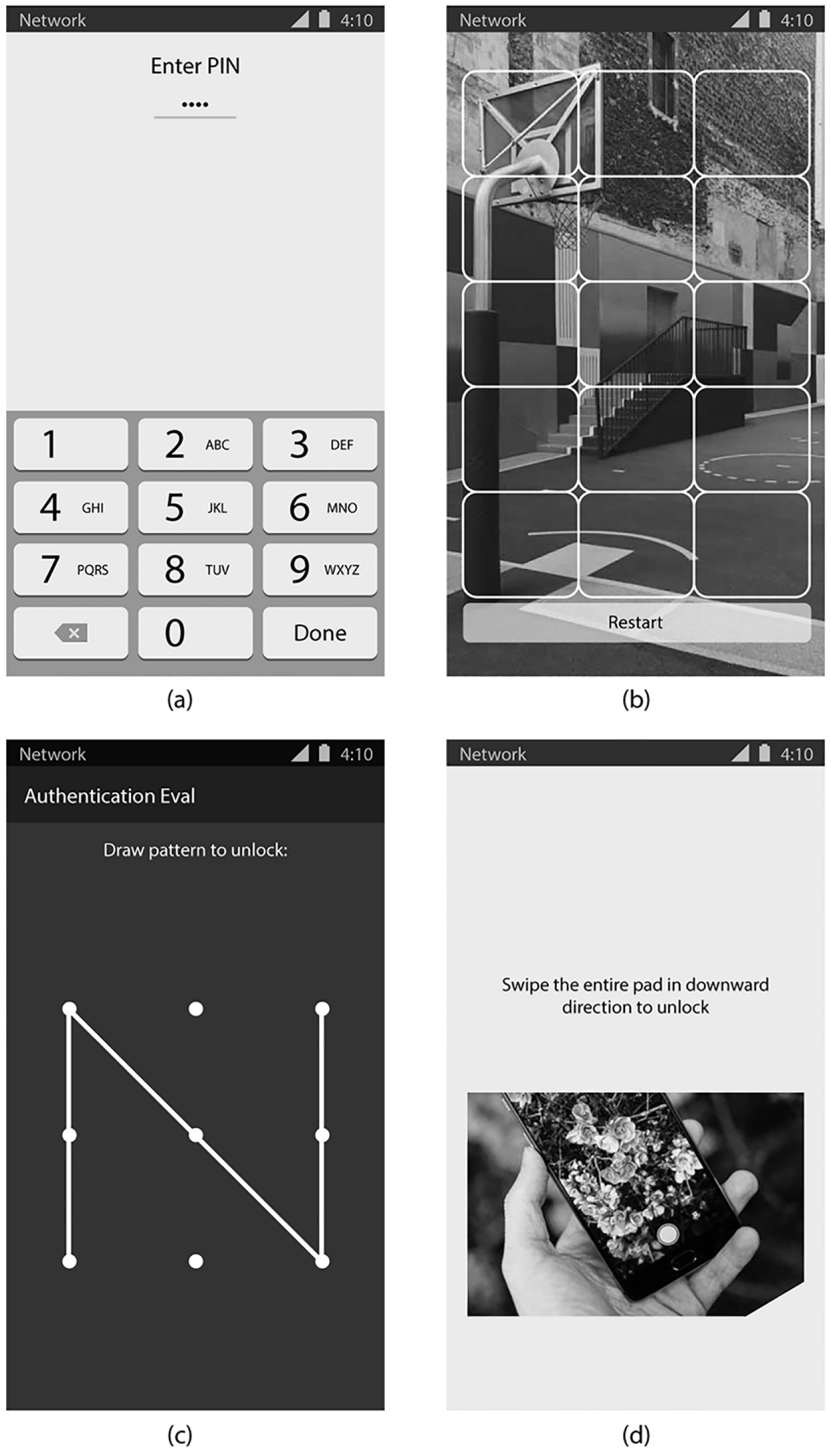
Authentication interfaces used in the evaluation: (a) PIN; (b) graphical password; (c) pattern-lock; (d) fingerprint.

We administered the test app using a Samsung S5 smartphone (Android OS, version 5.0). The Samsung device was used because it allows programmable interfaces for the fingerprint sensor. The test app took participants through the following four authentication techniques in random order (Fig 1):

1. *Four-digit PIN (knowledge-based)*: the participant entered a sequence of four numbers by tapping on a numeric keypad. Each participant created a sequence of four numbers that did not include four identical numbers (e.g., 1111) or numbers in ascending order (e.g., 1234).
2. *Pattern-lock (knowledge-based)*: the participant drew a pattern visualized as a line by connecting dots displayed in a 3 x 3 grid. Each participant created a pattern that adhered to standard Android restrictions where at least four unique dots must be connected and a dot must be used the first time it is passed over.
3. *Graphical password (knowledge-based)*: the participant selected a cell from a 3x5 grid superimposed on an image, and completed this five times using a five-image sequence. Participants selected images from an album of color images of natural landscapes, animals and humans.
4. *Fingerprint (biometric)*: the participant slid their finger across a physical “home button”. The fingerprint was compared to a database of the participant’s fingerprints that we captured in the initial configuration phase.

Through the app, we presented the four authentication interfaces (PIN, PATTERN, GRAPHICAL or FINGERPRINT) to participants in random order. Participants completed 20 authentication tasks, where they needed to successfully authenticate with a single option at least 20 times before they could move on to configure the next option. In other words, if they successfully authenticated 19 times and made one error, then they would need to make an additional attempt, for a total of 21 attempts. We also included a distractor task between each authentication attempt to reduce the confounding practice effect. The readings were adapted from national disease organization websites.

### Data collection

At baseline, participants completed a paper-based questionnaire about their experience with personal technology, security preferences, and demographics (S1 Questionnaire). As described above, for the test, participants entered each authentication measure 20 times while reading a health message between each entry. The test app captured data on whether the participant successfully authenticated on a task, the time to perform each successful authentication task (time spent on failed authentications was discarded), and the number of errors made on each task before successful authentication.

After testing an authentication option, participants completed the System Usability Scale (SUS) [19] as a validated measure of usability. Participants also used a 5-item Likert scale (1 = strongly agree, 2 = agree, 3 = neutral, 4 = disagree, 5 = strongly disagree) to rate their agreement that an option was tiring, annoying, and time consuming (S2 Questionnaire). Once all four authentication techniques were tested, participants rated each option on a scale of 0 (did not like at all) to 10 (liked very much), and ranked the techniques based on preference (1 = most preferred, 4 = least preferred) (S3 Questionnaire). Finally, after pilot testing, we modified the study protocol to include a semi-structured interview to explore what participants liked and disliked about each option.

### Outcome measures

Reliability and usability of the authentication techniques was measured by: 1) the proportion of time a participant was able to successfully authenticate on a single option (SUCCESS) over 20 attempts; 2) the mean time needed by a participant to successfully authenticate during each task (TIME); and 3) the mean number of errors a participant made before a successful authentication task (ERRORS).

### Data analysis

All statistical analyses were performed using IBM SPSS (Version 24). We summarized study demographics using descriptive statistics and compared participant SUCCESS, rankings and ratings across younger and older participants using t-tests, chi-square tests, and Mann-Whitney U tests. For participants who were able to successfully complete an authentication option, we used a mixed model analysis of variance (ANOVA) to compare TIME, ERRORS, and SUCCESS as well as the SUS across authentication techniques. The between subject factor was age, which had two levels (younger, older), and the within subject factor that was repeated was the authentication method, which had four levels (PIN, GRAPHICAL, PATTERN, FINGERPRINT). Qualitative data, including written explanations and interview data, were thematically hand coded and summarized to further explain quantitative data.

## Results

### Participants

A total of 102 participants were recruited, 43 being younger and 59 being older (Table 1). Overall, 62% were female (63/102), including 74% of young participants (32/43) and 52% of older participants (30/59). Further, 61% of participants were Caucasian (62/102), and 39% (40/102) represented a visible minority including 14% who identified as Chinese and 13% who identified as South Asian. Older participants were more likely to live with a chronic condition (χ^2^ = 34.65, *p* < 0.0005) and take prescription medications (χ^2^ = 22.67, *p* < 0.0005). Conditions in older adults included cardiovascular disease, arthritis, benign prostatic hypertrophy, Parkinson’s disease, acquired brain injury, fibromyalgia and osteoporosis, while chronic conditions in younger adults included anemia and migraines. Older participants also had a higher income (χ^2^ = 18.63, *p* = 0.001), less education (χ^2^ = 11.34, *p* = 0.045), and less ethnic diversity (χ^2^ = 47.00, *p* < 0.0005) than younger participants. All enrolled participants were found to have adequate health literacy (HLA score ≥8/18) and normal cognitive function (MoCA score ≥26/30) and there were no significant differences between age groups.

**Table 1:** Demographics of younger and older participant populations (N = 102).

	Age 18–30 (*N* = 43)	Age ≥ 50 (*N* = 59)
Age, mean (range)	24 (18–30)	67 (50–84)
Female, N (%)*	32 (74%)	31 (53%)
Highest Level of Education (%)*		
• High school & Trade School	3 (7)	8 (14)
• College	1 (2)	8 (14)
• University	39 (91)	43 (72)
Annual Income (%)*		
• <$20,000	10 (23)	0
• $20,000–49,000	4 (9)	12 (21)
• $50,000–79,999	6 (14)	9 (15)
• >$80,000	11 (26)	26 (44)
• Not reported	12 (28)	12 (20)
Ethnicity (%)*		
• Caucasian	12 (28)	51 (86)
• Aboriginal	0	1 (2)
• Black	0	1 (2)
• Arab	1 (2)	0
• Chinese	10 (23)	4 (6)
• South Asian	13 (30)	0
• Southeast Asian	2 (5)	0
• Korean	2 (5)	0
• Filipino	1 (2)	0
• Hispanic Latino	2 (5)	1 (2)
• Other	0	1 (2)
Daily technology use (%)		
• Computer	41 (98)	50 (89)
• Cellphone	2 (5)	12 (20)
• Smartphone*	43 (100)	31 (53)
• Tablet computer*	10 (23)	26 (44)
≥1 Chronic health conditions (%)*	5 (12)	42 (71)
≥ 1 Prescription medications (%)*	9 (21)	41 (70)
Health Literacy Assessment score	17.40 (1.37)	17.29 (1.60)
Montreal Cognitive Assessment score	28.91 (1.31)	29.02 (0.99)

*p < 0.05

In terms of prior technology use, most participants used a computer daily. All younger adults used a smartphone daily, compared to 53% (31/59) of older adults (χ^2^ = 5.18, *p* = 0.02). In contrast, 23% (10/43) of younger adults used a tablet computer regularly, while 44% (26/59) of older adults used a tablet daily (χ^2^ = 17.46, *p* = 0.001).

#### Overall usability and reliability

As seen in Table 2, all 102 participants attempted the authentication techniques and 87 participants were able to successfully authenticate into all 4 options. PATTERN had the fastest average authentication speed (3.44s), PIN and GRAPHICAL had the fewest average number of errors per attempt (0.02 errors/attempt) and participants had an average SUCCESS rate of over 98% using PIN, PATTERN and GRAPHICAL techniques. By comparison, the swipe-style FINGERPRINT on the Samsung 5 Android device was the slowest authentication option with an average TIME of 26.97s, an average of 1.46 ERRORS per attempt and a SUCCESS rate of 85% (described further in the Discussion).

**Table 2:** Success rate, time per authentication task, and errors per task PIN, PATTERN, GRAPHICAL and FINGERPRINT authentication techniques.

	Login Success Rate		Authentication Time (s)			Errors per Attempt (n)		
	n	%	Mean	Median	SD	Mean	Median	SD
PIN	100	98	4.71	3.86	2.43	0.02	0	0.04
Pattern	101	99	3.44	3.23	1.37	0.04	0	0.06
Graphical	102	100	6.76	6.14	2.88	0.02	0	0.06
Fingerprint	87	85	26.97	14.74	34.88	1.46	0.25	3.19

SD, standard deviation

For the 87 participants who successfully completed all four trials, TIME differed significantly between techniques, F (3, 258) = 37.40, *p* < 0.0005. Post hoc tests using the Bonferroni correction showed that all techniques were significantly different from one another, with PATTERN being 24 seconds faster than FINGERPRINT (mean difference (MD) = 23.98, standard error (SE) = 3.72, 95% confidence interval (CI) = (13.92, 34.04), *p* < 0.0005), 3 seconds faster than GRAPHICAL (MD = 3.20, SE = 0.28, 95% CI = (2.47, 3.95), *p* < 0.0005*)* and 1 second faster than PIN (MD = 1.14, SE = 0.23, 95% CI = (0.51, 1.77), *p* < 0.0005). PIN was also 2 seconds faster than GRAPHICAL (MD = 2.07, SE = 0.26, 95% CI = (1.37, 2.78), *p* < 0.0005). FINGERPRINT was over 20 seconds slower than all other techniques (*p* < 0.0005).

The number of ERRORS per task also significantly differed between options (F (3, 258) = 17.59, *p* < 0.0005). On average, FINGERPRINT had approximately one more error than all other options including PIN (MD = 1.45, SE = 0.34, 95% CI = (0.51, 2.34), *p* = 0.001), GRAPHICAL (MD = 1.44, SE = 0.34, 95% CI = (0.51, 2.37), *p* = 0.001), and PATTERN (MD = 1.43, SE = 0.34, 95% CI = (0.50, 2.35), *p* = 0.001). There were no other significant differences between options.

Finally, the rate of SUCCESS also differed significantly by task (F (3, 297) = 87.41, *p* < 0.0005). On average, FINGERPRINT had a success rate that was 8% lower than all other options including PIN (MD = 7.71, SE = 0.76, 95% CI = (5.65, 9.77), *p* = 0.001), GRAPHICAL (MD = 8.13, SE = 0.78, 95% CI = (6.02, 10.24), *p* = 0.001), and PATTERN (MD = 7.65, SE = 0.81, 95% CI = (5.46, 9.84), *p* = 0.001). There were no other significant differences between options.

#### Effects of age and chronic illness on usability and reliability

In terms of SUCCESS across age groups (Table 3), 100% of younger participants were able to complete 20 trials of the PIN, PATTERN and GRAPHICAL options and 98% were able to complete the trials of the FINGERPRINT option. By comparison, 100% of older participants were able to complete all required authentications for GRAPHICAL, 98% were able to complete PATTERN and PIN and 76% were able to complete FINGERPRINT.

**Table 3:** ANOVA comparing the average success rate, time per authentication task, and errors per for PIN, PATTERN, GRAPHICAL and FINGERPRINT authentication techniques (N = 86).

		All Techniques		Comparing Techniques Overall		
	N	Mean	95% CI	df	F	*p*-value
Authentication time (s)*	87	10.01	(8.12, 11.91)	(3, 258)	37.40	< 0.0005
Errors per attempt*	87	0.32	(0.16, 0.47)	(3, 258)	17.59	< 0.0005
Login success rate*	100	0.91	(0.89, 0.93)	(3, 297)	87.41	< 0.0005

*Only includes participants who successfully authenticated to all four authentication techniques

There were statistically significant two-way interactions between age and technique for each of the variables of TIME, ERROR and SUCCESS. For TIME, the statistical significance of a simple main effect was accepted at a Bonferroni-adjusted alpha level of 0.025. There was a statistically significant simple main effect of age for PIN (F (1, 82) = 17.95, *p* = <0.0005), PATTERN (F (1, 82) = 27.23, *p* = <0.0005), and GRAPHICAL (F (1, 82) = 12.61, *p* = 0.001), but not FINGERPRINT, (F (1, 82) = 3.74, *p* = 0.04). All pairwise comparisons were performed for statistically significant simple main effects. Bonferroni corrections were made with comparisons within each simple main effect that were considered to be a family of comparisons. On average based on pairwise comparisons, it took older adults two to three seconds longer to authenticate using the PIN (MD = 2.48, SE = 0.59, 95% CI = (1.32, 3.65), *p* = <0.0005), PATTERN (MD = 1.65, SE = 0.32, 95% CI = (1.02, 2.28), *p* = <0.0005) and GRAPHICAL (MD = 2.47, SE = 0.69, 95% CI = (1.08, 3.85), *p* = <0.0005). On further analysis, the statistical significance of a simple main effect of age for either ERROR or SUCCESS was not accepted at a Bonferroni-adjusted alpha level of 0.025.

Interactions with chronic illness were also investigated. Two-way interactions between chronic illness and technique were not statistically significant (*p* > 0.05). A three-way mixed ANOVA was run to understand the effects of age, chronic illness and authentication technique on each of TIME, ERROR and SUCCESS. None of the three-way interactions were statistically significant (*p* > 0.05).

#### Pre-test authentication experience

Most participants had experience using a PIN, simple password and secure password (Table 4). By contrast, younger participants had significantly more experience with PINs (χ^2^ = 69.44, *p* = 0.001), patterns (χ^2^ = 69.44, *p* = 0.001) and fingerprints (χ^2^ = 10.99, *p* = 0.001) and both groups had limited experience with graphical passwords. When asked about the types of authentication techniques used to unlock a computer, 67% (29/43) of younger participants reported using simple or secure passwords compared to only 44% of older adults (26/59). By comparison, the three most common authentication techniques used by younger participants on smartphones were PIN (33%), fingerprint (26%) and pattern (21%) compared to no password (29%) and PIN (17%) for older users.

**Table 4:** Participant pre-test experience and perceptions of authentication techniques (N = 102).

	Age 18–30 (*N* = 43)	Age ≥ 50 (*N* = 59)
Past experience (%)		
• 4-digit PIN*	42 (98)	51 (86)
• Simple password	30 (70)	38 (64)
• Secure password	41 (95)	52 (88)
• Pattern*	28 (65)	2 (3)
• Graphical password	9 (21)	7 (11)
• Fingerprint*	25 (58)	5 (9)
Forgets passwords (%)		
• Always	0 (0)	1 (1.7)
• Very often	6 (26)	9 (15)
• Sometimes	9 (39)	27 (46)
• Rarely	6 (26)	15 (25)
• Never	2 (9)	7 (12)
Writes down passwords* (%)		
• Always	2 (5)	24 (41)
• Very often	9 (21)	9 (15)
• Sometimes	7 (16)	12 (20)
• Rarely	15 (35)	9 (15)
• Never	10 (23)	5 (9)
Difficulty entering a password into smartphone or tablet (%)		
• Always	0 (0)	0 (0)
• Very often	1 (2)	3 (5)
• Sometimes	8 (19)	12 (20)
• Rarely	14 (32)	21 (36)
• Never	20 (47)	19 (32)
Agree/strongly agree it is “important to secure personal health information using a PIN or password” (%)	32 (74)	50 (85)
Typical authentication used for personal health information (%) PIN		
• Simple password	5 (11)	14 (24)
• Secure password	2 (5)	8 (14)
• Phrase password	20 (47)	21 (36)
• Graphical password	5 (12)	3 (5)
• Pattern-lock	0 (0)	0 (0)
• Fingerprint	0 (0)	1 (2)
• Nothing used	4 (8)	0 (0)
Preferred authentication used for personal health information (%)		
• PIN	8 (19)	16 (27)
• Simple Password	1 (2)	8 (14)
• Secure Password	8 (19)	12 (20)
• Phrase Password	2 (5)	3 (5)
• Graphical password	0 (0)	4 (7)
• Pattern-lock	3 (7)	2 (3)
• Fingerprint	16 (38)	12 (20)

*p < 0.05

While there was no significant difference in how often the different age groups reported forgetting passwords (χ^2^ = 0.73, *p* = 0.95), older participants were significantly more likely to write passwords down compared to younger participants (χ^2^ = 21.11, *p* <0.0005). In their written explanations, younger participants reported that the most common strategies used to remember passwords included resetting the password while older participants were more likely to look the password up before resetting it. For older participants, common places to look up a password included written notes, an electronic spreadsheet or asking a spouse whereas younger participant would store it in the browser. When creating a password, younger and older participants both reported using similar approaches including using different variations of a root password (39% vs. 31%). Older participants were also more likely to report using a common term such as the name of grandchild or an important date (25% vs 9%).

#### Post-test authentication experience

**Fig 2:**
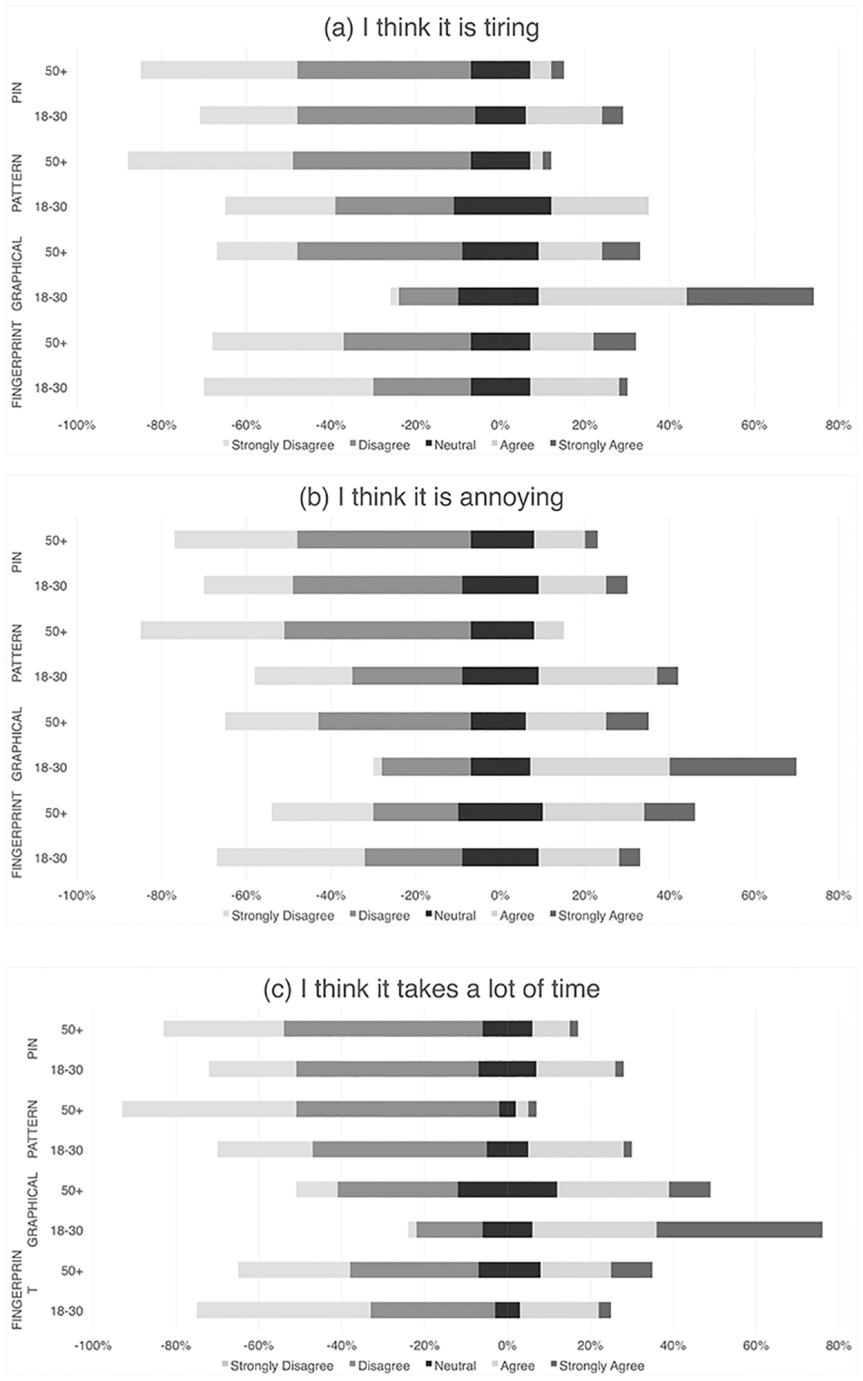
Participants’ agreement after testing PIN, PATTERN, GRAPHICAL, and FINGERPRINT that the (a) Scheme is tiring; (b) Scheme is annoying; and (c) Scheme is time consuming (N = 100).

As seen in the Likert plot (Fig 2), older participants did not feel that FINGERPRINT was any more or less tiring, annoying or time consuming compared to younger participants (*p* > 0.05). In terms of PIN, older participants found it to be less tiring (t = -2.04, *p* = 0.04) but felt it was similarly annoying and time consuming compared to younger participants. Older participants also found PATTERN to be less annoying (t = -3.16, *p* = 0.002), and time consuming (t = −3.22, *p* = 0.002) than younger participants. Similarly, older participants found the GRAPHICAL option to be less tiring (t = −5.16, *p* <0.0005), annoying (t = −4.29, *p* < 0.0005), and time consuming (t = −3.80, *p* < 0.005).

**Fig 3:**
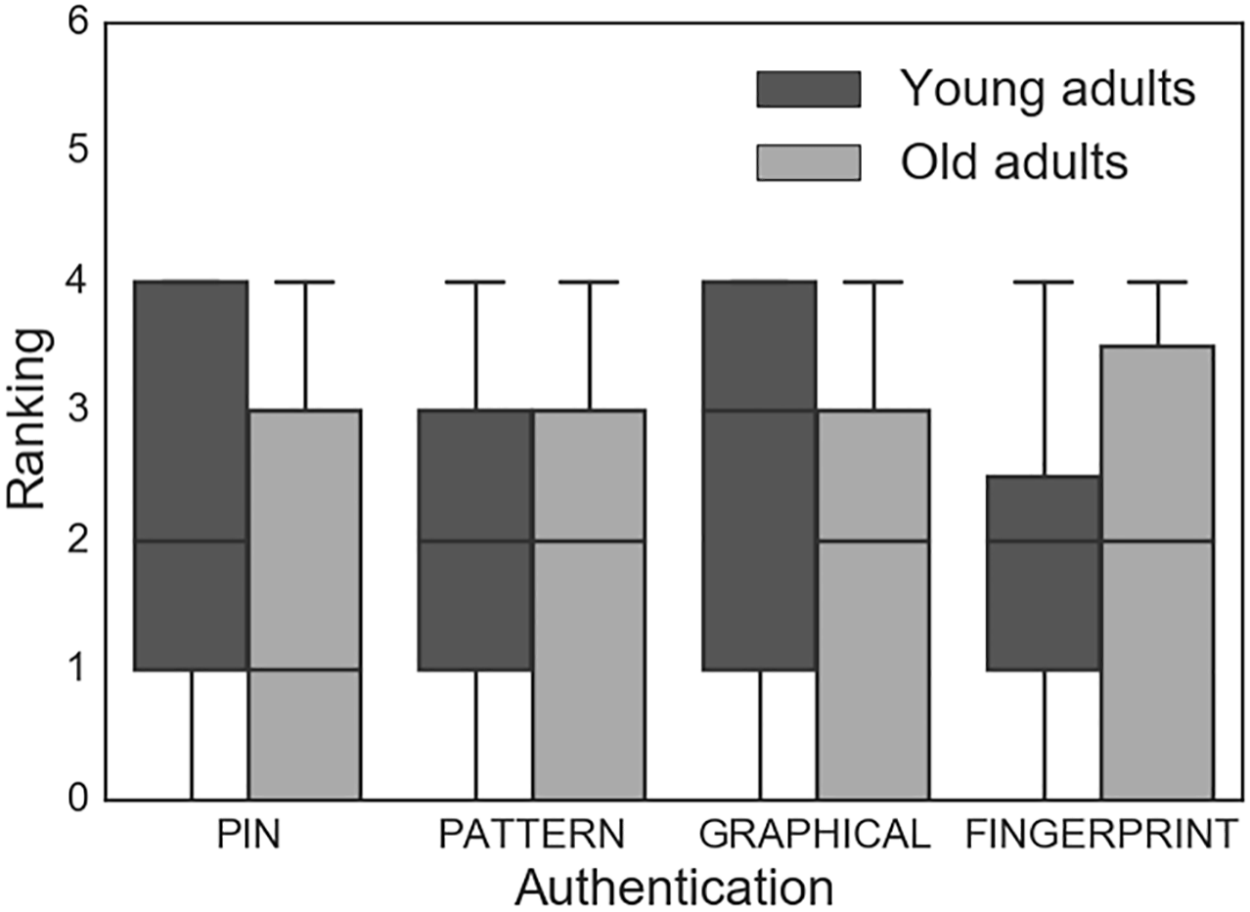
Participant rankings of the PIN, PATTERN, GRAPHICAL and FINGERPRINT authentication techniques from 1 (most preferred) to 4 (least preferred) (N = 56).

Participants were asked to rank their preferred authentication option in order of preference, with the most preferred option being ranked as a 1 and the least preferred option being ranked as a 4 (Fig 3). Of the 78 participants who ranked the options, the option with the best mean ranking was FINGERPRINT (2.33), followed by PATTERN (2.35), GRAPHICAL (2.65), and PIN (2.6). To identify any differences between the rankings of the authentication techniques, we conducted a multivariate linear regression by including all four rankings of (PIN, PATTERN, GRAPHICAL, and FINGERPRINT), and found that age groups have no significant effect on the participants’ ranking/preferences.

**Fig 4:**
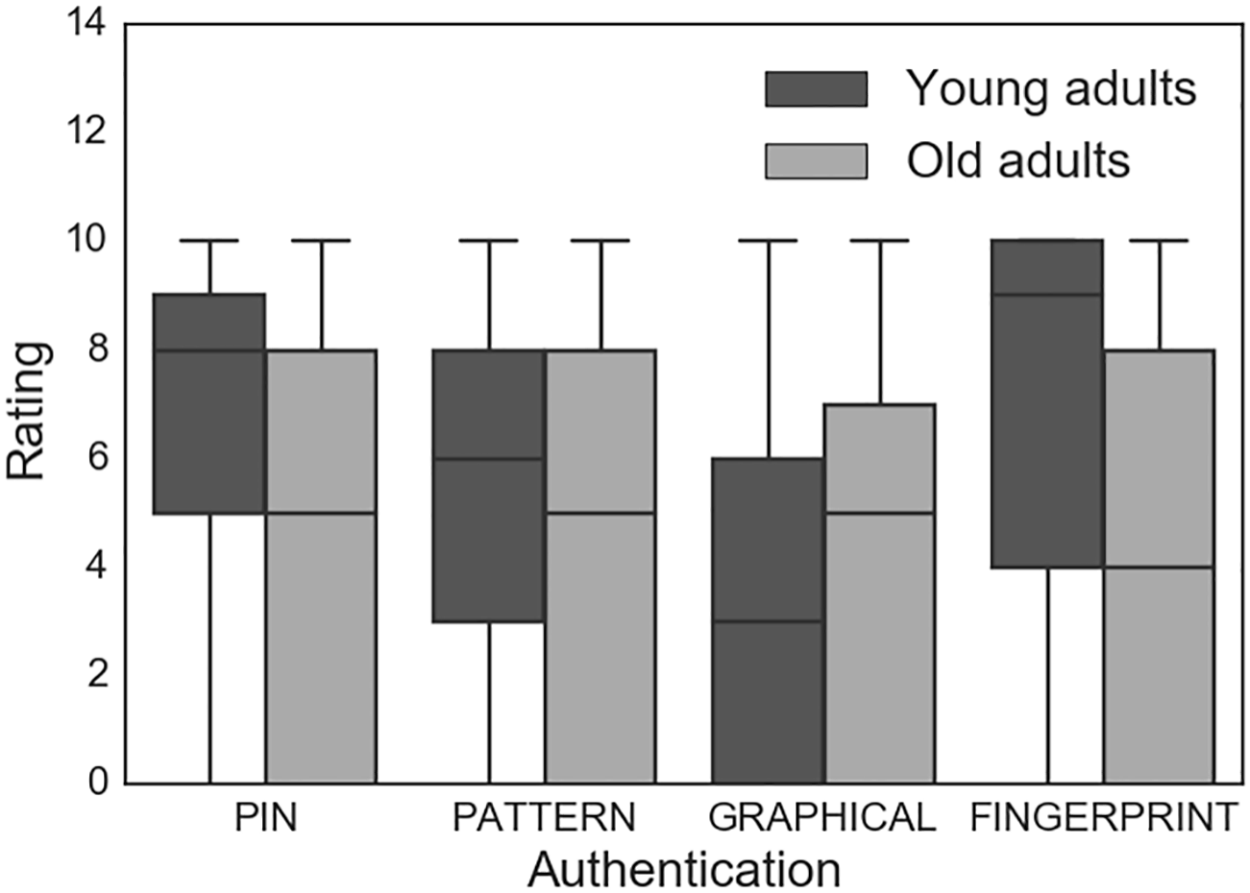
Participant ratings of how much they liked the PIN, PATTERN, GRAPHICAL and FINGERPRINT authentication techniques from 0 (did not like at all) to 10 (liked very much) (N = 56).

Similarly, participants were asked to rate how much they liked the techniques (0 = do not like at all, 10 = like very much). According to the mean rankings, the most highly rated option was PIN (7.15), followed by FINGERPRINT (6.94), PATTERN (6.63) and GRAPHICAL (5.12) (Fig 4). Using the Wilcoxon signed rank tests, GRAPHICAL was rated lower than all other options, including PIN (Z = -3.91, *p* < 0.0005), FINGERPRINT (Z = -3.30, *p* = 0.001), and PATTERN (Z = -3.58, *p* < 0.0005). There were no significant differences in how older and younger adults rated authentication options (p > 0.05).

#### System usability scale (SUS)

**Fig 5:**
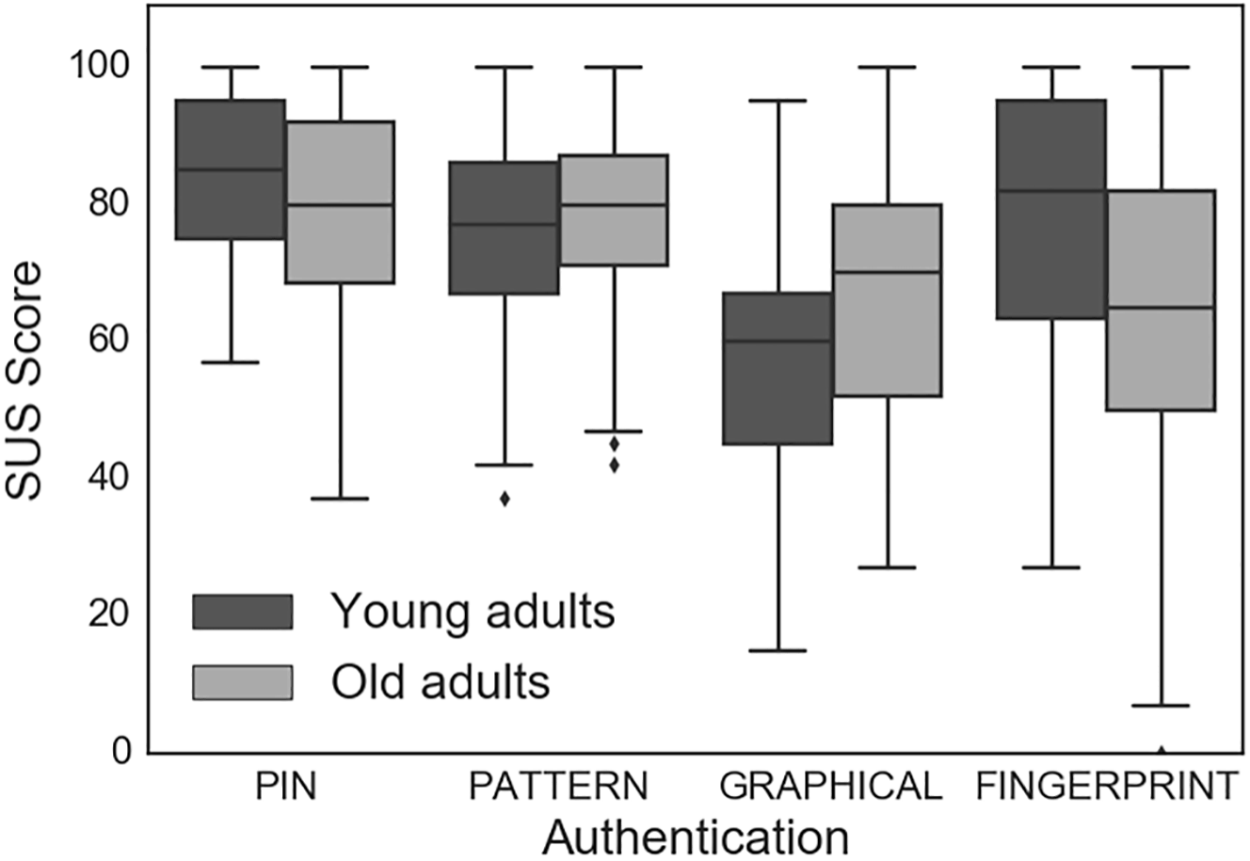
Boxplot of systems usability scale score for PIN, PATTERN, GRAPHICAL and FINGERPRINT for younger and older adults (N = 56).

As seen in Fig 5, we used the mixed model ANOVA to determine that there were significant differences in mean SUS scores between authentication techniques (F (3, 282) = 23.26 *p* < 0.0005) and between age groups (F (3, 270) = 388.08, *p* < 0.0005). Post hoc tests using the Bonferroni correction showed that for all participants, the SUS score for PIN was 18 points higher than GRAPHICAL (MD = 17.76, SE = 2.12, 95% CI = (12.05, 23.47), *p* < 0.0005) and 11 points higher than FINGERPRINT (MD = 11.40, SE = 2.49, 95% CI = (4.70, 18.09), *p* < 0.0005). The SUS score for PATTERN was also 15 points higher than GRAPHICAL (MD = 15.34, SE = 2.10, 95% CI = (9.67, 21.01), *p* < 0.0005) and 9 points higher than FINGERPRINT (MD = 8.97, SE = 2.75, 95% CI = (1.56, 16.39), *p* = 0.009). Finally, compared to younger participants, older participant SUS scores were 14 points lower for FINGERPRINT (MD = -13.50, SE = 2.78, *p* = 0.004) and F 4.28, *p* = 0.04) and 12 points higher for GRAPHICAL (MD = 11.94, SE = 3.47, *p* = 0.001).

## Discussion

In this multidisciplinary study of four possible authentication techniques for mHealth apps, we found that older adults take two to three seconds longer than younger adults to authenticate using the PIN, pattern and graphical password passwords and 20 seconds longer with the swipe-style fingerprint. Older users are also less likely to successfully authenticate using a fingerprint. We also found that, in both groups, the pattern-lock had the fastest average authentication speed (3.44s), the PIN and PATTERN had the fewest average number of errors per attempt (0.02) and participants were able to login using the PIN, pattern and graphical password at least 98% of the time.

Notably, the swipe-style fingerprint was the slowest authentication option with an average time of 27 seconds, an average of 1.5 errors per attempt and a successful login rate of 85%. The poor performance of the fingerprint authentication scheme may be unique to how the study device’s (Samsung S5) fingerprint scanner works. To authenticate on the study device’s fingerprint scanner, users are required to slide a finger with a firm, smooth swipe across the home button. On the other hand, Apple’s Touch ID only requires its users to tap or touch the home button to unlock the device. It is unclear whether the high number of failed authentication attempts is a result of the study device’s requirement of sliding the finger in the exact way it requires. Issues may also have been related to dry skin, shaking hands and poorer coordination. In terms of usability, the graphical password was rated the lowest overall and the fingerprint and graphical password were rated lower than the PIN and pattern with the SUS.

Our findings are consistent with other authentication research that has found the PIN and pattern-lock to be quick and usable options. Researchers have evaluated text-based passwords to show that while they are perceived to be secure, the constrained virtual keyboards on smartphones are a bottleneck in their adoption [20]. An evaluation of PIN and pattern-lock schemes shows that the PIN outperforms the pattern-lock in terms of input speed and error rate, however, users favor pattern-lock and rate it better in terms of ease-of-use and likeability [21]. Researchers have evaluated the usability of Apple’s Touch ID (fingerprint) and Android’s Face Unlock and found that while users found both schemes easy to use, Touch ID performed poorly when the hands were dirty and Face Unlock failed under poor lightening conditions [14].

Our research also shows that research with older adults on authentication techniques for web services may not be transferable to mobile devices. For example, Renaud and Ramsay [22] have proposed and evaluated a graphical password scheme for web services. Contrary to our findings, their evaluations showed that their proposed scheme was better in terms of usability and error rate than PINs. Another draw-a-secret based graphical password scheme for online services has been proposed by Sreeramareddy *et al*. [23]. Their evaluations indicate that their scheme was accurate and usable and could be used as an alternate form of authentication for older adults.

Despite their popularity, PIN and password-based authentication systems provide relatively weak security protection compared with “something a person has,” such as a swipe card, or “something a person is,” such as a fingerprint [24]. Researchers predict that biometric measures such as face-recognition and fingerprints have the potential to be more secure than PINs and passwords [25]. In our study, the swipe-style fingerprint option scored lower on all measures of usability, but was still preferred by participants overall. This growing preference is consistent with other research that has shown that, while PINs and passwords continue to be the favored option for people over age 40, those under 40 prefer biometric authentication [26]. This suggests that we should see a consumer-led shift in the authentication choices.

This study demonstrates how healthcare and security experts should be engaged in mHealth design to identify when and how sensitive information should be secured. Health informatics and security researchers are also encouraged to test authentication measures with all ages. Given the shortcomings of current authentication protocols, multi-factor authentication schemes continue to be recommended for highly sensitive material, meaning the user provides two or more of the three “know/has/is” combinations. In the US, the HIPAA Security Guidance report advises using two-factor authentication [27]. In an mHealth app, that could include a password and a fingerprint, for example. However, in practice that may prove too onerous for patients with complex health needs, especially given our findings that older adults have a high failure rate with swipe-style fingerprint authentication.

There are a few limitations that need to be considered in the interpretation of our study. First, we included an atypical study population when compared to a general patient population that also includes vulnerable patients with low levels of health and technical literacy. The goal of our study was to compare the typical user testing population with a population that is more representative of healthcare. Thus, we cannot assume that younger populations who live with chronic illness would perform in a similar fashion to the comparatively healthy young adults in our study. Second, it is critical to remember that age may not be the sole determinant of differences between groups and a larger sample size is likely required to identify the different characteristics that influence user experience. Third, this is a cross-sectional study that was limited to a brief testing period. Future research using longitudinal study design is needed to better understand how older adults adapt to authentication techniques over time. For example, patterns or text passwords may be easier to remember and use in the long-term. Finally, we did not test a simple text password in the study as we deemed it sufficiently similar to a 4-digit PIN.

In conclusion, we found that on a mobile device, the PIN and pattern-lock authentication performed well across age groups. Older adults took slightly longer to authenticate to the PIN, pattern and graphical passwords, but the PIN and pattern-lock techniques were still highly rated for usability in both groups. Future research on authentication methods should include more diverse samples of study participants to support wide adoption of future options.

## Supporting information

S1 Questionnaire Demographics questionnaire. (DOCX)

S2 Questionnaire Usability questionnaire. (DOCX)

S3 Questionnaire Exit questionnaire. (DOCX)
