# Supplementary material for: Evaluating authentication options for mobile health applications in younger and older adults
Source: PLoS One. 2018 Jan 4;13(1):e0189048. doi: 10.1371/journal.pone.0189048 (PMC5754080; doi:10.1371/journal.pone.0189048)
Supplement: S1 Questionnaire — (DOCX) [file pone.0189048.s001.docx]

**Please complete the following questionnaire. If you have difficulty, the research coordinator can assist you. The following questions will help us understand more about you and your experience with passwords.**

1. The following is a list of password options. Select all options that you have used before:

- PIN
  - E.g., 1234 or 0984 or 2098 or 2093
- Simple password, or dictionary word, such as
  - E.g., sunny, today, person, school, Susan
- Secure password of 8+ digits including a small letter, big letter, number and/or symbol
  - E.g., AsoineN1%
- Secure password remembered using a phrase
  - E.g., A really good grade is 90% = Arggi90%
- Image-based passcode: selecting the right images or the right spots on provided images to authenticate yourself;
- Fingerprint
- Pattern Lock
  - E.g.


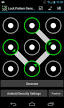


2. Do you own a computer (desktop or laptop)?

- Yes
- No ---**skip to question 6**

3. How often do you use your computer?

- Daily
- Weekly
- Monthly
- Rarely

4. What kind of password do you need to enter to unlock your computer?

- PIN
- Simple password
- Secure password
- Secure password remembered using a phrase
- Pattern lock
- Image based passcode
- Fingerprint
- I don’t need to enter a password, pattern, image based passcode or fingerprint

5. Do you need to enter a password to unlock a specific software program on your computer (specify:)?

6. Do you own a regular cellphone such as a flip phone, not including smartphone?

- Yes
- No ---**skip to question 9**

7. How often do you use your regular cellphone?

- Daily
- Weekly
- Monthly
- Rarely

8. What kind of password do you need to enter to unlock your regular cellphone?

- PIN
- Simple password
- Secure password
- Secure password remembered using a phrase
- Pattern lock
- Image based passcode
- Fingerprint
- I don’t need to enter a password, image based passcode, pattern or fingerprint

9. Do you own a smartphone such as an Apple iPhone, Samsung Galaxy or a Blackberry Z, etc?

- Yes
- No ---**skip to question 13**

10. How often do you use your smartphone?

- Daily
- Weekly
- Monthly
- Rarely

11. What kind of password do you need to enter to unlock your smartphone?

- PIN
- Simple password
- Secure password
- Secure password remembered using a phrase
- Pattern lock
- Image based passcode
- Fingerprint
- I don’t need to enter a password, image based passcode, pattern or fingerprint

12. Do you need to enter a password to unlock a specific software program on your smartphone (specify)?

13. Do you own a handheld tablet computer such as an Apple iPad or a Google Nexus or Samsung Galaxy TAB?

- Yes
- No ---**skip to question 17**

14. How often do you use your tablet?

- Daily
- Weekly
- Monthly
- Rarely

15. What kind of password do you need to enter to unlock your tablet?

- PIN
- Simple password
- Secure password
- Secure password remembered using a phrase
- Pattern lock
- Image based passcode
- Fingerprint
- I don’t need to enter a password, image based passcode, pattern or fingerprint

16. Do you need to enter a password to unlock specific software program on your tablet (specify)?

17. Do you use the same password for multiple software programs on your personal computer, smartphone and/or tablet computer?

- Yes
- No
- I don’t own a computer, smartphone or tablet computer

18. How often do you write down your passwords and store them somewhere else?

- Always
- Very Often
- Sometimes
- Rarely
- Never

19. How often do you forget your password(s)?

- Always
- Very Often
- Sometimes
- Rarely
- Never—**skip to question 21**

20. What do you do when you forget a password? Explain:

21. What do you do to help remember passwords? Explain:

22. How often do you have difficulty entering/typing your password into your smartphone or tablet computer?

- Always
- Very Often
- Sometimes
- Rarely
- Never
- I don’t own a smartphone or tablet computer

**Note: The following questions help us to ensure that we include a variety of people in our study. You can choose to not answer the question if you’d like.**

23. What is your education (select all that apply)?

- Below high school
- High school
- Trade school
- College
- University
- Graduate Degree (MA, PhD)
- Professional Degree (MD, MBA)

24. What is your **annual** household income (the combined income of all individuals living in your home)?

- Less than $20,000
- $20,000-$49,999
- $50,000-$79,999
- More than $80,000
- I don’t know/refuse to answer

25. What is your gender?

- Man
- Woman
- Other (e.g. Transgender)

26. Which of the following best represents your ethnicity (select all that apply):

- Caucasian
- Aboriginal
- Black
- Arab
- Chinese
- Japanese
- Korean
- West Asian
- South Asian
- Southeast Asian
- Filipino
- Hispanic/Latino
- Other (Explain):

27. Do you have chronic health conditions in the past three months?

- Yes (Please specify: _________________________________________)
- No

28. Are you on prescription medication?

- Yes (Please specify: _________________________________________)
- No

29. Do you take dietary supplements?

- Yes (Please specify: _________________________________________)
- No

****Thank you for completing this questionnaire. The research coordinator will now perform a short assessment of your health literacy. Please notify the research coordinator that you are ready to begin the assessment.**

**Word**

| 1. Kidney | 4. Nutrition | 7. Alcoholism | 10. Dose | 13. Directed | 16. Diagnosis |
| --- | --- | --- | --- | --- | --- |
| 2. Occupation | 5. Miscarriage | 8. Pregnancy | 11. Hormones | 14. Nerves | 17. Hemorrhoids |
| 3. Medication | 6. Infection | 9. Seizure | 12. Abnormal | 15.Constipation | 18. Syphilis |

**Health Literacy Assessment Script**

Research Coordinator: The following is a short assessment of your health literacy. (A score between 0 and 14 suggests the examinee has low health literacy). Look at the list of words you have. Read the first word out loud. Next, I’ll read two words and I’d like you to tell me which of the two words is more similar to or has a closer association with the word you just read. If you don’t know, please say ‘I don’t know’. Don’t guess.

Next, read the second word… (Continue until the list is complete).

| **Stem** | **Key or Distracter** | | **Don't know** |
| --- | --- | --- | --- |
| 1. Kidney | __Urine | __Fever | __Don’t know |
| 2. Occupation | __Work | __Education | __Don’t know |
| 3. Medication | __Instrument | __Treatment | __Don’t know |
| 4. Nutrition | __Healthy | __Soda | __Don’t know |
| 5. Miscarriage | __Loss | __Marriage | __Don’t know |
| 6. Infection | __Plant | __Virus | __Don’t know |
| 7. Alcoholism | __Addiction | __Recreation | __Don’t know |
| 8. Pregnancy | __Birth | __Childhood | __Don’t know |
| 9. Seizure | __Dizzy | __Calm | __Don’t know |
| 10. Dose | __Sleep | __Amount | __Don’t know |
| 11. Hormones | __Growth | __Harmony | __Don’t know |
| 12. Abnormal | __Different | __Similar | __Don’t know |
| 13. Directed | __Instruction | __Decision | __Don’t know |
| 14. Nerves | __Bored | __Anxiety | __Don’t know |
| 15. Constipation | __Blocked | __Loose | __Don’t know |
| 16. Diagnosis | __Evaluation | __Recovery | __Don’t know |
| 17. Hemorrhoids | __Veins | __Heart | __Don’t know |
| 18. Syphilis | __Contraception | __Condom | __Don’t know |
