# Supplementary material for: Evaluating authentication options for mobile health applications in younger and older adults
Source: PLoS One. 2018 Jan 4;13(1):e0189048. doi: 10.1371/journal.pone.0189048 (PMC5754080; doi:10.1371/journal.pone.0189048)
Supplement: S2 Questionnaire — (DOCX) [file pone.0189048.s002.docx]

**Usability and Learnability of Authentication Measures Survey**

**Session: PIN***

A reminder on the types of Authentication Measures:

1. PIN
   - E.g., 1234 or 0984 or 2098 or 2093
2. Simple password or your choosing
   - E.g., sunny, today, person, school, Susan
3. Secure password of 8+ digits including a letter, number and/or symbol
   - E.g., AsoineN1%
4. Secure password remembered using a phrase
   - E.g., A really good grade is 90% = Arggi90%
5. Image Based Passcode: select the right spot on provided images in certain order;
6. Fingerprint
7. Pattern lock

- E.g.


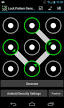


*(Same questionnaire used for Pattern, Fingerprint, and Image)

1. In your daily life, do you agree: “it is important to secure your **personal health information** using a PIN or Password”?

- Strongly Agree
- Agree
- Neutral
- Disagree
- Strongly Disagree

1. When you want to protect your **personal health information** on a computer or mobile device, what type of password do you typically use?

- PIN
- Simple password
- Secure password
- Secure password remembered using a phrase
- Image based passcode
- Pattern lock
- Fingerprint
- I don’t use a password, image based passcode, pattern or fingerprint

1. Given the choice, when you want to protect your **personal health information** on a computer or mobile device, what type of password do you most prefer to use on a mobile device?

- PIN
- Simple password
- Secure password
- Secure password remembered using a phrase
- Image based passcode
- Pattern lock
- Fingerprint

**The following questions are about your experience using the PIN (the following questions were also used for GRAPHICAL, PATTERN, and FINGERPRINT).**

1. How secure do you think a PIN is **compared to no password** to unlock your mobile device?

- Much more secure
- Somewhat more secure
- Not more or less secure
- Somewhat less secure
- Much less secure
- I don’t know

1. How secure do you think a PIN is **compared to the type of password you typically use** in your daily life?
   - Much more secure
   - Somewhat more secure
   - Not more or less secure
   - Somewhat less secure
   - Much less secure
   - I don’t know
2. Rate your agreement with the following statements about the PIN method?

|  | Strongly  Agree | Agree | Neutral | Disagree | Strongly Disagree |
| --- | --- | --- | --- | --- | --- |
| I think entering PIN takes a lot of time |  |  |  |  |  |
| I think PIN method is annoying |  |  |  |  |  |
| I think the PIN method is tiring |  |  |  |  |  |

1. Rate your agreement with the following statements about the **PIN method**?

| # |  | Strongly Agree | Agree | Neutral | Disagree | Strongly Disagree |
| --- | --- | --- | --- | --- | --- | --- |
| *1* | I think I would use the PIN frequently |  |  |  |  |  |
| *2* | I found the PIN unnecessary complex |  |  |  |  |  |
| *3* | I found the PIN easy to use |  |  |  |  |  |
| *4* | I think I would need the support of a technical person to be able to use the PIN method |  |  |  |  |  |
| *5* | I thought there was too much inconsistency in using the PIN method |  |  |  |  |  |
| *6* | I would imagine most people would learn to use the PIN very quickly |  |  |  |  |  |
| *7* | I found the PIN very cumbersome to use |  |  |  |  |  |
| *8* | I felt confident using the PIN |  |  |  |  |  |
| *9* | I needed to learn a lot of things before I could get going with the PIN |  |  |  |  |  |
| *10* | I found the PIN was well integrated with the various functions of the task. |  |  |  |  |  |
