# Supplementary material for: Evaluating authentication options for mobile health applications in younger and older adults
Source: PLoS One. 2018 Jan 4;13(1):e0189048. doi: 10.1371/journal.pone.0189048 (PMC5754080; doi:10.1371/journal.pone.0189048)
Supplement: S3 Questionnaire — (DOCX) [file pone.0189048.s003.docx]

1. On a scale of 0 to 10, rate how much you liked each method that you tried (0=did not like at all; 10 liked very much).

|  | Did not like at all | | | | Neutral | | | | | Liked very much | | | |
| --- | --- | --- | --- | --- | --- | --- | --- | --- | --- | --- | --- | --- | --- |
|  | 0 | 1 | 2 | 3 | | 4 | 5 | 6 | 7 | | 8 | 9 | 10 |
| PIN |  |  |  |  | |  |  |  |  | |  |  |  |
| Picture |  |  |  |  | |  |  |  |  | |  |  |  |
| Pattern |  |  |  |  | |  |  |  |  | |  |  |  |
| Fingerprint |  |  |  |  | |  |  |  |  | |  |  |  |

1. Considering everything together, including security, reliability, accessibility, etc., please rank the 4 password strategies you tried during the study from most preferred to least preferred.

(Most preferred)

(1)

(2)

(3)

(4)

(Least preferred)

***Please pass this back to the researcher***

*The following questions will be asked by the research coordinator:*

1. **(If there is mismatch ranking between question 1 and 2): I noticed that you ranked the [e.g., PIN] highest but you liked the [e.g., fingerprint] more. Why is that?**
2. **What did you like and dislike about each method you tried:**

|  | Like | Dislike |
| --- | --- | --- |
| PIN |  |  |
| Picture |  |  |
| Pattern |  |  |
| Fingerprint |  |  |

1. **What are some scenarios where a particular scheme would not work in your life?**
2. **Is there anything else that you’d like to add?**
